# Enhanced Benefits of Prone Positioning Combined with Lung Recruitment Maneuver in Patients with COVID-19 and Non-COVID-19 ARDS: A Secondary Analysis of a Randomized Clinical Trial

**DOI:** 10.3390/jcm14248822

**Published:** 2025-12-13

**Authors:** Lan Lan, Yuenan Ni, Yubei Zhou, Ping Li, Faping Wang, Fengming Luo

**Affiliations:** 1 Department of Respiratory and Critical Care Medicine, West China Hospital, Sichuan University, Chengdu 610041, China; lanlanay@163.com (L.L.); niyuenan0305@scu.edu.cn (Y.N.); zybhh1004@163.com (Y.Z.); liping13258136705@163.com (P.L.); wangfpscu@gmail.com (F.W.); 2 State Key Laboratory of Respiratory Health and Multimorbidity, West China Hospital, Sichuan University, Chengdu 610041, China

**Keywords:** acute respiratory distress syndrome, prone position, recruitment maneuver, recruitability

## Abstract

**Background:** Early reports highlighted unique features of COVID-19-associated ARDS. The combination of prone position (PP) and positive end-expiratory pressure (PEEP)-induced lung recruitment maneuver (LRM) has demonstrated efficacy in enhancing oxygenation and improving outcomes in patients with ARDS, but it remains unknown whether there is a difference between COVID-19 ARDS and non-COVID-19 ARDS. **Method:** This study is a secondary analysis of a previously conducted randomized controlled trial. Patients with moderate to severe ARDS were consecutively enrolled during the study period (June–December 2023). After initiation of PP, patients received a PEEP-induced LRM followed by 12 h of daily PP. The interventions were repeated at least three times over the subsequent 3 days. Clinical outcomes, respiratory mechanics, and electrical impedance tomography (EIT) results were evaluated. **Results:** Twenty-eight patients were included in the final analysis, half of whom were infected with COVID-19 (50%). The PEEP-induced LRM led to greater improvement in oxygenation among COVID-19 ARDS than non-COVID-19 ARDS (∆PaO_2_/FiO_2_ ratio 90.5 mmHg vs. 65.5 mmHg, *p* < 0.05). Based on EIT measurement, compared with the non-COVID-19 ARDS group, PEEP-induced LRM resulted in a greater increase in ventilation distribution, mainly in the dorsal regions of interest 4 (ROI 4) ventilation distribution (∆ROI4 4.5% vs. 1.0%, *p* = 0.01) and in dorsal regional ventilation (∆dorsal regional ventilation 10.0% vs. 5.5%, *p* = 0.04) in the COVID-19 ARDS group. **Conclusions:** Compared to typical ARDS, PEEP-induced LRM combined with PP may be more effective in enhancing oxygenation in COVID-19-related ARDS.

## 1. Background

Acute respiratory distress syndrome (ARDS) presents as acute respiratory failure with refractory hypoxemia and bilateral chest opacities, arising from a variety of pulmonary and extrapulmonary insults [1]. COVID-19-associated ARDS is a unique subtype of ARDS, with significant overlap but notable differences from typical ARDS. Due to collapse, regional atelectasis, flooding, or consolidation, the functional lung area is reduced, particularly observed in the dorsal-dependent lung regions, while the ventral non-dependent regions preserve normal aeration [2]. In typical ARDS, the primary site of damage is the alveolar space, especially in pulmonary-originated ARDS. In contrast, COVID-19 ARDS primarily originates from the vascular side, disturbing the ventilation–perfusion matching, which could be the primary mechanism of hypoxemia in the early phase of COVID-19 ARDS [3]. Most patients admitted to the intensive care unit (ICU) with severe COVID-19 meet the diagnostic criteria for ARDS and require invasive mechanical ventilation. Assessing respiratory mechanics and lung recruitability can provide essential information for optimizing ventilator management [4].

Prone positioning, applied for critically ill patients since the 1970s, is recommended for ARDS treatment due to its benefits, including enhanced ventilation-perfusion matching, re-aeration of dorsal lung regions, and facilitating alveolar recruitment in the lower lobes and dorsal areas [5]. Electrical impedance tomography (EIT) provides a non-invasive, real-time visualization of lung regional ventilation distribution [6]. By applying high PEEP and airway pressure, LRM restores aerated lung volume, further improving compliance and oxygenation in ARDS. Additionally, higher PEEP is less likely to cause regional hyperinflation in the prone position [7]. Prone positioning and the application of PEEP-induced LRM are well-established strategies contributing to improving oxygenation and ventilation–perfusion matching in ARDS. However, the interaction between prone positioning and PEEP-induced LRM remains unclear, particularly in patients with COVID-19 ARDS, where the recruitment ability and vascular response may differ from those in non-COVID-19 ARDS. Previous physiological studies have demonstrated that the response of recruitment and hemodynamic effects to PEEP may be more heterogeneous in COVID-19 ARDS than in non-COVID-19 ARDS, suggesting that ventilation optimization strategies should consider these pathophysiologic distinctions [8].

Our previous study reported that PEEP-induced LRM, combined with prone positioning, acts synergistically in improving oxygenation and ventilation distribution in moderate to severe ARDS [9]. However, we did not differentiate between patients with COVID-19 and non-COVID-19 ARDS. Thus, we conducted a secondary analysis focusing on these two groups to clarify the differential impacts of these interventions. Few studies have explored the potential interactions between PP and PEEP-induced LRM in ARDS, with limited high-quality evidence supporting this hypothesis in COVID-19 ARDS. Additionally, few have directly compared the effects between typical ARDS and COVID-19 ARDS. Therefore, we sought to characterize the effects of prone positioning combined with PEEP-induced LRM in COVID-19 ARDS and directly compare them with those of typical ARDS.

## 2. Methods

### 2.1. Study Design

This study is a secondary observational analysis derived from a prospective, single-center randomized controlled trial (ChiCTR2300072905, registered on 27 June 2023, chictr.org.cn) conducted in West China Hospital, Sichuan University (Chengdu, China), protocol code 2023—Review (248) and date of approval, 24 February 2023. Ethical approval was obtained from the institutional review board, and written informed consent was provided by each participant, or their legally authorized representative when applicable, prior to inclusion in the study.

### 2.2. Patient Selection

Eligible participants in the original trial met the following criteria: (1) age ≥ 18 years, (2) mechanical ventilation for >24 h, and (3) diagnosis of moderate or severe ARDS according to the Berlin definition. Patients were excluded for (1) pneumothorax, severe barotrauma; (2) hemodynamic instability (vasopressor increase >30% within 6 h or norepinephrine >0.5 μg/kg/min); (3) severe COPD; (4) PaO_2_/FiO_2_ < 60 mmHg; or (5) suspected intracranial hypertension >18 mmHg. For this secondary analysis, only patients who were randomized to the PP combined with LRM group, completed the full intervention protocol, and had complete EIT recordings were included (*n* = 28).

### 2.3. Ventilation and Intervention Strategy

**Figure 1:**
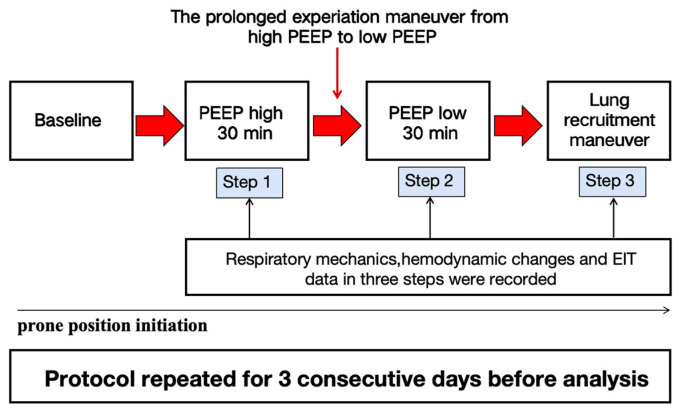
Flowchart of the study.

Mechanically ventilated patients with moderate to severe ARDS were enrolled and randomized to prone positioning (PP) alone or PP combined with PEEP-induced lung recruitment maneuvers (LRM). For this secondary analysis, only patients in the PP with LRM group were included and were further stratified by COVID-19 status. The intervention involved performing LRM one hour after PP initiation, repeated at least three times over 3 days. All patients received standardized volume-controlled ventilation and were kept under deep sedation and neuromuscular blockade. LRM was performed using a three-step protocol: PEEP at 18 cmH_2_O for 30 min, decreased to 8 cmH_2_O for 30 min, then increased again to 18 cmH_2_O. Respiratory mechanics and hemodynamics were recorded during each phase (Figure 1). Oxygenation (PaO_2_/FiO_2_) and lung recruitability, assessed by the recruitment-to-inflation (R/I) ratio, were evaluated one hour post-intervention. The intervention procedures and flow chart of patients included in the trial are detailed in the Supplementary Materials (Figures S1 and S2).

### 2.4. Electric Impedance Tomography

Electrical impedance tomography (EIT; PulmoVista 500, Dräger Medical, Lübeck, Germany) was used to continuously monitor regional ventilation. The electrode belt was positioned around the thorax at the fourth or fifth intercostal space. Image reconstruction was performed from impedance variations (ΔZ) to visualize pixel-level ventilation distribution. The ventilated lung regions were divided into ventral (non-dependent) and dorsal (dependent) zones for quantitative analysis. At each recording point, a stable period of approximately 30 consecutive breaths was selected for analysis.

### 2.5. Statistical Analysis

Data are presented as numbers (percentages) for categorical variables and medians (first and third quartiles) for continuous variables. Statistical analysis was performed using SPSS 26 (IBM) and Prism 9.0 (GraphPad Software). Normal distribution was confirmed by the Shapiro–Wilk test. Mann–Whitney U tests or Fisher’s exact tests were used for categorical data comparisons. Qualitative data were analyzed using the chi-square test or Fisher’s exact test. Statistical significance was set at *p* ≤ 0.05 for all tests. The sample size was defined by the number of patients who completed the intervention protocol in the original RCT (*n* = 28). Consistent with prior EIT-based physiological studies, this sample size is considered adequate for exploratory physiological analyses.

## 3. Results

In this analysis, we included 28 consecutive patients with ARDS admitted between 27 June 2023 and 1 January 2024. As shown in Table 1, we prospectively include 14 patients with COVID-19 ARDS (50%) and 14 with non-COVID-19 ARDS, including bacterial ARDS (28.6%) and extrapulmonary origin ARDS (21.4%). The median R/I ratio did not differ significantly between the COVID-19 and non-COVID-19 ARDS groups (0.7 [0.4–0.8] vs. 0.6 [0.3–0.8], *p* = 0.44). The PEEP-induced LRM led to greater improvement in PaO_2_/FiO_2_ among COVID-19 ARDS subjects than non-COVID-19 ARDS (∆PaO_2_/FiO_2_ ratio 90.5 [63.0, 159.0] mmHg vs. 65.5 [24.5, 94.2] mmHg, *p* < 0.05). No significant change was found in other respiratory and hemodynamic parameters.

**Figure 2:**
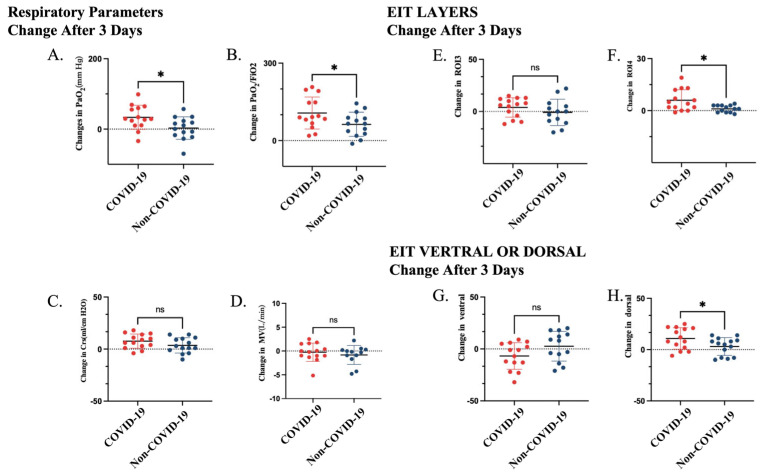
Changes in respiratory parameters and EIT-derived regional ventilation 3 days after intervention in patients with COVID-19 and non-COVID-19 ARDS. (**A**) Change in PaO_2_ (ΔPaO_2_). (**B**) Change in PaO_2_/FiO_2_ ratio. (**C**) Change in respiratory system compliance (ΔCrs). (**D**) Change in minute ventilation (ΔMV). (**E**) Change in tidal ventilation in ROI 3 (ΔTV ROI 3). (**F**) Change in tidal ventilation in ROI 4 (ΔTV ROI 4). (**G**) Change in ventral regional ventilation. (**H**) Change in dorsal regional ventilation. * *p* < 0.05; ns, not significant. Abbreviations: PaO_2_, arterial partial pressure of oxygen; FiO_2_, fraction of inspired oxygen; Crs, respiratory system compliance; MV, minute ventilation.

Based on EIT assessment, PEEP-induced LRM promoted a more pronounced dorsal redistribution of tidal ventilation in COVID-19 ARDS. Specifically, the change in the dorsal ROI4 layer was higher in the COVID-19 group than in non-COVID-19 ARDS (∆ROI4 4.5 [1.5, 12.3] % vs. 1.0 [−1.0, 3.0] %, *p* = 0.01). Similarly, the dorsal regional ventilation fraction increased more prominently in COVID-19 ARDS (10.0 [1.0, 21.3] % vs. 5.5 [−8.0, 8.5] %, *p* = 0.04), indicating enhanced recruitment of dependent lung regions (Table 1, Figure 2).

## 4. Discussion

In this study, we explored the effect of PEEP-induced LRM performed in the prone position among patients with COVID-19 ARDS and non-COVID-19 ARDS. We found that PEEP-induced LRM combined with the prone position resulted in significantly greater improvement in oxygenation and ventilation distribution, particularly in the dorsal regions, as assessed by EIT, in patients with COVID-19 ARDS compared with non-COVID-19 ARDS.

The application of PEEP and the recruitability of lung tissue in COVID-19 ARDS remain debated. The predominant pathophysiological mechanisms in COVID-19 ARDS are highly atypical and markedly different from those in typical ARDS, in which hypoxemia arises from a true right-to-left shunt (perfusion of non-aerated tissue) and/or a low V/Q ratio (perfusion of poorly ventilated lung regions) [10]. Fossali et al. reported that PP improves lung recruitment and V/Q matching in COVID-19 ARDS by reducing atelectrauma and anterior hyperinflation [11]. Our findings align with this, demonstrating that PP and LRM may synergistically enhance oxygenation and ventilation uniformity in COVID-19 ARDS, likely through enhanced dorsal lung recruitment. Additionally, lower-pressure LRM may achieve recruitment goals with fewer adverse effects, such as barotrauma or circulatory depression.

Currently, there is no evidence demonstrating that the combination of prone positioning and LRM is more effective in COVID-19 ARDS than typical ARDS. PaO_2_/FiO_2_, as the primary measure of ARDS severity, has since been demonstrated to be an independent risk factor for mortality in adults with ARDS and was also a significant predictor of survival in patients with moderate-to-severe ARDS [12,13]. Optimizing oxygenation during prone positioning can reduce further lung damage and prevent multi-organ failure due to hypoxia, which has been associated with shorter ICU stays and improved survival rates [14]. Oxygenation after prone positioning significantly differed between ICU survivors and non-survivors, with improvement observed in ARDS survivors [15]. As reported by some studies, COVID-19 ARDS was characterized by higher lung compliance dissociated with oxygenation and had consistently worse oxygenation variables following a period of mechanical ventilation [16], which may imply that improving oxygenation is essential for long-term management of prolonged intubation patients during the progression of COVID-19 ARDS.

Lung recruitability, defined as the recruitment-to-inflation (R/I) ratio, was calculated as the proportion between the compliance of the recruited lung volume and the total respiratory system compliance at low PEEP. No significant difference in R/I was observed between COVID-19 and non-COVID-19 ARDS, in line with previous findings of heterogeneous recruitability in COVID-19 ARDS [17]. In our study, greater oxygenation improvement after PEEP-induced LRM occurred without corresponding changes in baseline R/I, suggesting that oxygenation may not directly reflect the overall recruitability. The possible mechanism may be explained as follows. The increase in the dorsal ventilation ratio indicates the enhancement of regional recruitment, while the R/I ratio measured by the ventilator reflects the compliance of the entire respiratory system, but this indicator may not reflect the local recruitment pattern. Furthermore, the original “baby lung” area may experience partial overexpansion, and at lower levels of PEEP, the alveoli will open periodically, which may result in a decrease in overall assessable expandability.

COVID-19 ARDS is recognized as a heterogeneous condition encompassing distinct physiological phenotypes. Early observations suggested two major subtypes: type L, characterized by relatively preserved compliance, low elastance, and limited recruitability, and type H, showing low compliance, high elastance, and greater potential for recruitment [16,18]. These phenotypes may evolve dynamically during the progression of pneumonia, with some patients transitioning from type L to type H as the disease advances. Subsequent physiological studies have indicated that most COVID-19 ARDS cases demonstrate substantial or variable lung recruitability [19]. Given this heterogeneity, the physiological response to lung recruitment maneuvers during prone positioning may vary across individuals. Further, prospective studies are warranted to clarify the efficacy and safety of PEEP-induced LRM combined with prone positioning in different COVID-19 ARDS phenotypes and to refine personalized ventilatory management strategies.

Our study has several limitations. First, we described a small sample size of patients with COVID-19 ARDS, which limits the generalizability of the findings in a broader ARDS population. Second, we analyzed respiratory and EIT data only after three days, without assessing longitudinal changes. Third, as a secondary analysis, we did not stratify or adjust patients according to proposed COVID-19 ARDS phenotypes, and the results should therefore be interpreted as exploratory.

## 5. Conclusions

This study shows that, among patients with moderate–severe ARDS treated with PEEP-induced LRM during PP, COVID-19 ARDS has greater improvement in oxygenation than non-COVID-19 ARDS. EIT findings further demonstrated a more enhanced dorsal redistribution of ventilation in COVID-19 ARDS, despite similar baseline R/I ratios, suggesting that global indices of recruitability may not fully reflect etiology-specific ventilation responses. The clinical implications of these physiology-based differences, and the potential role of combined PP and LRM in personalized management of COVID-19–related ARDS, should be evaluated in larger, prospectively phenotyped trials. Further research is warranted to determine the optimal timing, pressure settings, and patient subgroups most likely to benefit from this therapy.

## Figures and Tables

**Table 1:** Characteristics and delta change in enrolled patients.

	COVID-19 (14)	Non-COVID-19 (14)	*p* Value
**Demographic data**			
Age, years	61 [45.0, 80.0]	55.0 [40.0, 68.0]	0.40
Female sex, *n* (%)	3.0 (21.4%)	1.0 (7.1%)	0.28
BMI, median (Q1–Q3), kg/m^2^	22.3 [21.7, 24.9]	23.3 [20.5, 27.6]	0.82
APACHE II at ICU admission, median (Q1–Q3)	27.0 [21.0, 31.0]	23.0 [22.0, 26.0]	0.16
RASS at ICU admission, median (Q1–Q3)	−4.0 [−4.0, −3.0]	−4.0 [−4.0, −3.0]	0.48
Days intubated before Randomization, median (Q1–Q3)	2.0 [1.0, 2.3]	1.5 [1.0, 2.3]	0.24
Hemodynamics, median (Q1–Q3)			
Heart rate (bpm), median (Q1–Q3)	92.5 [77.5, 106.0]	113.5 [89.3, 132.5]	0.11
SpO_2_ (%), median (Q1–Q3)	97.7 [96.4, 99.5]	97.1 [94.5, 98.6]	0.11
Mean arterial pressure (mmHg), median (Q1–Q3)	90.0 [81.0, 106.0]	94.5 [77.3, 109.5]	0.43
**Baseline ventilator settings, median (Q1–Q3)**			
Tidal volume (mL)	450.0 [400.0, 460.0]	480.0 [425.0, 480.0]	0.69
Respiratory rate, breaths/min	21.0 [18.0, 24.0]	20.0 [16.0, 27.5]	0.95
PEEP (cmH_2_O)	10.0 [10.0, 12.0]	10.0 [10.0, 12.0]	0.81
Crs (mL/cmH_2_O)	34.5 [23.8, 43.5]	35.5 [29.3, 40.5]	0.93
Ppeak (cmH_2_O)	28.0 [26.5, 30.5]	26.5 [24.0, 28.5]	0.12
Pplat (cmH_2_O)	25.0 [22.5, 27.0]	22.5 [21.0, 25.0]	0.11
Pmean (cmH_2_O)	17.0 [15.8, 19.3]	16.5 [14.0, 18.0]	0.12
Raw (cmH_2_O)	9.5 [5.0, 13.0]	8.50 [7.00, 14.5]	0.72
Driving pressure (cmH_2_O)	15.0 [11.8, 16.0]	11.50 [10.0, 13.5]	0.10
**Arterial blood gas, median (Q1–Q3)**			
pH	7.3 [7.3, 7.4]	7.4 [7.3, 7.4]	0.73
PaO_2_ (mmHg)	94.9 [76.7, 125.2]	91.0 [74.9, 131.5]	0.81
PaCO_2_ (mmHg)	44.4 [35.0, 54.6]	43.6 [38.9, 48.4]	0.92
HCO_3_^−^ (mmol/L)	22.5 [20.7, 30.9]	24.3 [21.2, 25.8]	0.47
PaO_2_/FiO_2_ (mmHg)	103.6 [80.3, 135.1]	123.1 [95.4, 145.9]	0.21
**Mortality**			
28 days mortality	6 (42.9%)	7 (50.0%)	0.71
90 days mortality	9 (64.2%)	7 (50.0%)	0.45
Overall mortality	9 (64.2%)	10 (71.2%)	0.69
**Length of stay, days**			
Intensive care unit, days	22.0 [17.0, 39.0]	23.0 [13.0, 28.0]	0.92
Hospital, days	34.0 [21.0, 55.0]	42.0 [24.0, 70.0]	0.77
**Lung recruitability**			
R/I	0.7 [0.4, 0.8]	0.6 [0.3, 0.8]	0.44
**Change after 3 days**			
∆tidal volume (mL)	−2.0 [−24.0, 16.8]	−15.5 [−53.5, 33.5]	0.70
∆MV (L/min)	−0.1 [−1.1, 1.5]	−0.2 [−1.5, 0.3]	0.47
∆C_rs_ (mL/cmH_2_O)	7.5 [2.5, 14.3]	4.5 [−0.5, 10.8]	0.14
∆PaO_2_ (mmHg)	29.4 [10.8, 61.2]	5.7 [−18.4, 26.2]	0.02 *
∆PaO_2_/FiO_2_ (mmHg)	90.5 [63.0, 159.0]	65.5 [24.5, 94.2]	0.04 *
**EIT data**			
∆TV ROI 1 layers (%)	−9.0 [−15.3, 0.3]	−1.0 [−5.5, 2.5]	0.02 *
∆TV ROI 2 layers (%)	−4.0 [−8.3, 8.8]	4.0 [−4.0, 13.0]	0.32
∆TV ROI 3 layers (%)	5.5 [−4.5, 12.3]	0.0 [−10.5, 7.3]	0.36
∆TV ROI 4 layers (%)	4.5 [1.5, 12.25]	1.0 [−1.0, 3.0]	0.01 *
∆ventral of tidal image region (%)	−9.0 [−22.3, 0.8]	4.0 [−11.8, 15.8]	0.07
∆dorsal of tidal image region (%)	10.0 [1.0, 21.3]	5.5 [−8.0, 8.5]	0.04 *

COVID-19, coronavirus disease 2019; ICU, intensive care unit; APACHE II, Acute Physiology and Chronic Health Evaluation; RASS, Richmond Agitation-Sedation Scale; BMI, Body Mass Index; bpm, beats per minute; SpO_2_, Pulse Oxygen Saturation; PaO_2_, Partial Pressure of Arterial Oxygen; HCO_3_^−^, bicarbonate; PaCO_2_, Partial Pressure of Arterial Carbon Dioxide; PaO_2_/FiO_2_, Ratio of Arterial Oxygen Partial Pressure to Inspired Oxygen Fraction; PEEP, positive end-expiratory pressure; Ppeak, peak airway pressure; Pplat, plateau pressure; Pmean, mean airway pressure; Crs, respiratory system compliance; Raw, airway resistance. ROI, region of interest. * *p* < 0.05.

## Data Availability

All data analyzed and discussed in the framework of this study are included in this published article.

## Supplementary Materials

The following supporting information can be downloaded at: https://www.mdpi.com/article/10.3390/jcm14248822/s1, STROBE Statement—checklist of items that should be included in reports of observational studies; S1: Study population and enrollment process; S2: The periods of recruitment and follow-up; Figure S1: STROBE flow diagram isllustrating the study protocol. A total of 28 patients were included in the final analysis (14 with COVID-19 ARDS and 14 with non-COVID-19 ARDS). Reasons for exclusion are detailed in the figure; Figure S2: Flow chart of patients included in the trial.

## Abbreviations

ARDS	Acute respiratory distress syndrome
ABG	Arterial blood gas
ALI	Acute Lung Injury
BMI	Body Mass Index
Crs	Respiratory system compliance
COVID-19	Coronavirus disease 2019
EIT	Electrical impedance tomography
HCO_3_^−^	Bicarbonate
ICU	Intensive care unit
IQR	Interquartile Range
LRM	Lung recruitment maneuver
MV	Minute Ventilation Volume
PaCO_2_	Partial Pressure of Arterial Carbon Dioxide
PaO_2_	Partial Pressure of Arterial Oxygen
PaO_2_/FiO_2_	Arterial Oxygen Tension to Inspired Oxygen Fraction Ratio
PEEP	Positive end-expiratory pressure
Ppeak	Peak Pressure
Pplat	Plateau pressure
Pmean	Mean airway pressure
Raw	Airway resistance
RASS	Richmond Agitation-Sedation Scale
R/I Ratio	Recruitment-to-Inflation Ratio
ROI	Region of interest
SpO_2_	Oxygen Saturation as Measured by Pulse Oximetry
TIV	Tidal Impedance Variation
TV/Vt	Tidal volume
VILI	Ventilator-Induced Lung Injury
PBW	Predicted Body Weight
